# Structural variants in linkage disequilibrium with GWAS-significant SNPs

**DOI:** 10.1016/j.heliyon.2024.e32053

**Published:** 2024-05-28

**Authors:** Hao Liang, Joni C. Sedillo, Steven J. Schrodi, Akihiro Ikeda

**Affiliations:** a Department of Medical Genetics, University of Wisconsin-Madison, Madison, WI, USA; b Computation and Informatics in Biology and Medicine, University of Wisconsin-Madison, Madison, WI, USA; c McPherson Eye Research Institute, University of Wisconsin-Madison, Madison, WI, USA

**Keywords:** GWAS, Structural variants, Genome-wide significance, Linkage disequilibrium, Disease gene mapping

## Abstract

With the recent expansion of structural variant identification in the human genome, understanding the role of these impactful variants in disease architecture is critically important. Currently, a large proportion of genome-wide-significant genome-wide association study (GWAS) single nucleotide polymorphisms (SNPs) are functionally unresolved, raising the possibility that some of these SNPs are associated with disease through linkage disequilibrium with causal structural variants. Hence, understanding the linkage disequilibrium between newly discovered structural variants and statistically significant SNPs may provide a resource for further investigation into disease-associated regions in the genome. Here we present a resource cataloging structural variant-significant SNP pairs in high linkage disequilibrium. The database is composed of (i) SNPs that have exhibited genome-wide significant association with traits, primarily disease phenotypes, (ii) newly released structural variants (SVs), and (iii) linkage disequilibrium values calculated from unphased data. All data files including those detailing SV and GWAS SNP associations and results of GWAS-SNP-SV pairs are available at the SV-SNP LD Database and can be accessed at ‵https://github.com/hliang-SchrodiLab/SV_SNPs. Our analysis results represent a useful fine mapping tool for interrogating SVs in linkage disequilibrium with disease-associated SNPs. We anticipate that this resource may play an important role in subsequent studies which investigate incorporating disease causing SVs into disease risk prediction models.

## Highlights

• Exhaustive, genome-wide correlation results between GWAS-significant SNPs and SVs.
• Findings provide SV information necessary for fine-mapping GWAS signals in humans.
• Quarriable online repository available.

## 1 Introduction

Large genomic imbalances can significantly disrupt important functional elements in the human genome including chromatin structure, noncoding RNAs, protein-coding sequence, and gene regulation [[1], [2], [3]]. These changes can substantially alter phenotypes and potentially drive a wide-array of disease and disease-related traits. These changes can therefore drive substantial phenotypic effects, including those effects that impact the risk of disease. Indeed, structural variants (SVs) have been associated with several clinical traits including schizophrenia [4], cardiometabolic physiology [5], amyotrophic lateral sclerosis [6], low density lipoprotein levels [7], and neurodevelopmental disorders [8]. However, short-read sequencing technology has limited ability to detect SVs [9]. To address this limitation, a study of 32 human genomes was recently conducted by the Human Genome Structural Variation Consortium (HGSVC) using a combination of long-read Pacific Biosciences whole genome sequencing and Strand-seq technologies, identifying 107,590 SVs across the genome [10]. The authors noted that 68 % of these SVs were not discovered by short-read sequencing. In our study, we sought to determine which single nucleotide polymorphisms (SNPs), exceeding genome-wide significance levels in genome-wide association studies (GWAS) were in high linkage disequilibrium with newly identified SVs. By doing so, these results generate specific hypotheses concerning SVs as causal variants which could drive correlated SNPs to exhibit strong disease association. Hence, this work can serve as a resource to investigate disease-association at SVs not interrogated in GWAS which may be driving disease signals.

The ability to utilize newly discovered SVs as potential causal variants to be verified in experimental studies can reveal unexpected molecular pathogenic mechanisms as they may disrupt functional motifs not identified through SNP data. In addition to the role of SVs in fine mapping of disease loci to elucidate the molecular pathogenesis of complex diseases, causal SVs can be incorporated in risk prediction models such as polygenic risk scores to improve disease classification, thereby advancing precision medicine [11].

## 2 Materials and methods

**Fig. 1:**
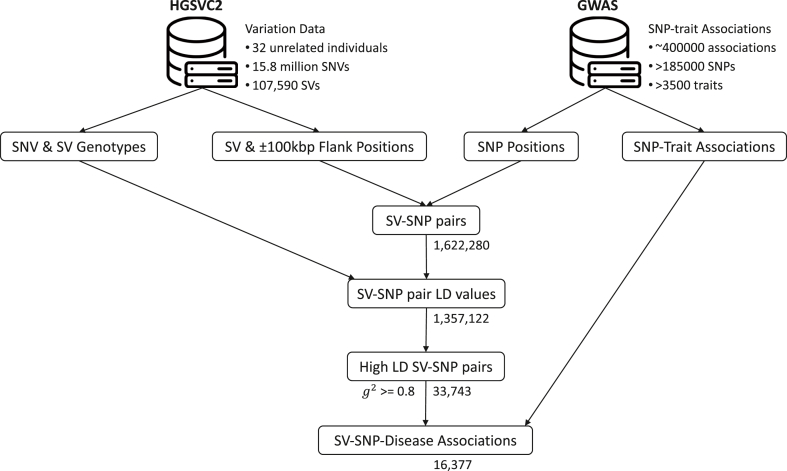
Overall Workflow of the Study. The study used several steps to identify SV-SNP-Disease associations: (i) SNV and GWAS-significant SNP data are merged by chromosome and position; (ii) Within the merged dataset, SV-GWAS SNPs were identified and pairwise linkage disequilibrium values were calculated; (iii) Those SV-GWAS SNP pairs with $g^{2}\geq 0.80$ were obtained; and (iv) the information with disease association and linkage disequilibrium were retained for the final database.

To construct the database of GWAS-significant SNPs (p < 5E-08) in high linkage disequilibrium with these new SVs, SNPs were obtained from the GWAS catalog [12] noting the position (assembly GRch38/hg38) and alleles. SV location and alleles were downloaded from the HGSVC data portal [13]. To reduce the computational effort, a restriction was placed on the physical distance between GWAS SNPs and SVs prior to calculating linkage disequilibrium. This distance was set to 100kbp flanking the endpoints of the SV. The 100kbp distance was selected to cover the majority of variants in high linkage disequilibrium around causal sites as historical recombination events reduce the region associated with disease to chromosomal segments somewhat smaller than 100kbp in human populations with large population sizes if we define the region as the physical distance until the standard linkage disequilibrium measure r^2^ decays, on average, to <0.20 from an initial site [14,15]. It is important to note that there is variability in the appropriate region size that is a function of population size, population demographics, natural selection, disease effect size at the causal site, recombination frequency in the genomic region, and the sample size of the studies [[16], [17], [18], [19]]. That said, to reduce the computational expense, 100kbp was deemed appropriate for this study. Future studies investigating the linkage disequilibrium patterns with newly discovered genetic variants may expand this size limit to potentially uncover additional highly correlated genetic markers. Following the restriction of the analysis to 100kbp from the endpoints of the SV, using the unphased data, linkage disequilibrium was then calculated on the HGSVC samples using the approach described below. GWAS SNP-SV pairs that exceeded a squared correlation coefficient of 0.80 were included in the database. Fig. 1 shows the overall design of the study with initial numbers of SNPs, traits, and SVs, and numbers of SNP-SV pairs that pass the physical distance and linkage disequilibrium filters.

Perl (v5.32.1) code was used to preprocess original files, including file format conversion, GWAS association extraction and sample counting. Code written in R (v4.1.3) was used to calculate linkage disequilibrium and p-values. Bedtools (v2.30.0) was also used to extract SNPs located within the regions of SVs and their upstream/downstream 100kbp flanking regions.

Our aim was to calculate pairwise linkage disequilibrium (LD) between each SV and nearby GWAS-significant SNPs without a gametic phasing step, for our goal was focused on addressing the hypothesis that a SV might be causally driving disease predisposition and therefore it is simply the correlation between SNP and SV pairs that is of primary interest, rather than understanding the haplotype frequencies. Hence, to calculate the pairwise LD, an estimator of the squared correlation coefficient was used on unphased data. Although a minor improvement, the use of unphased genotype data for two-site correlation calculations avoids error propagation derived from haplotype estimation inaccuracy [20]. The SVs and SNPs studied are biallelic. Denote the pair of alleles segregating at an SV as $A_{1}$ and $A_{2}$. Similarly denote the pair of alleles segregating at a SNP as $B_{1}$ and $B_{2}$. Further denote the number of the nine double genotypes in a sample of individuals as:

$$x_{1}=num\left(A_{1}A_{1}B_{1}B_{1}\right)$$

$$x_{2}=num\left(A_{1}A_{1}B_{1}B_{2}\right)$$

$$x_{3}=num\left(A_{1}A_{1}B_{2}B_{2}\right)$$

$$x_{4}=num\left(A_{1}A_{2}B_{1}B_{1}\right)$$

$$x_{5}=num\left(A_{1}A_{2}B_{1}B_{2}\right)$$

$$x_{6}=num\left(A_{1}A_{2}B_{2}B_{2}\right)$$

$$x_{7}=num\left(A_{2}A_{2}B_{1}B_{1}\right)$$

$$x_{8}=num\left(A_{2}A_{2}B_{1}B_{2}\right)$$

$$x_{9}=num\left(A_{2}A_{2}B_{2}B_{2}\right)\text{.}$$

Let $N=\sum_{i=1}^{9}x_{i}$.

Setting the numerical values for each individual carrying a specific genotype as

$$A_{1}A_{1}=1$$

$$A_{1}A_{2}=2$$

$$A_{2}A_{2}=3$$

$$B_{1}B_{1}=1$$

$$B_{1}B_{2}=2$$

$$B_{2}B_{2}=3\text{,}$$

we then define the genotypic squared correlation coefficient (Pearson's correlation coefficient squared) as.

$g^{2}=\frac{{\left[\mu_{XY}-\left(\mu_{X}\mu_{Y}\right)\right]}^{2}}{\left\{\left[\mu_{X^{2}}-{\left(\mu_{X}\right)}^{2}\right]\left[\mu_{Y^{2}}-{\left(\mu_{Y}\right)}^{2}\right]\right\}}$ ; where

$$\mu_{XY}=\frac{1}{N}\left(x_{1}+2x_{2}+3x_{3}+2x_{4}+4x_{5}+6x_{6}+3x_{7}+6x_{8}+9x_{9}\right)$$

$$\mu_{X}=\frac{1}{N}\left(x_{1}+x_{2}+x_{3}+2x_{4}+2x_{5}+2x_{6}+3x_{7}+3x_{8}+3x_{9}\right)$$

$$\mu_{Y}=\frac{1}{N}\left(x_{1}+2x_{2}+3x_{3}+x_{4}+2x_{5}+3x_{6}+x_{7}+2x_{8}+3x_{9}\right)$$

$$\mu_{X^{2}}=\frac{1}{N}\left(x_{1}+x_{2}+x_{3}+4x_{4}+4x_{5}+4x_{6}+9x_{7}+9x_{8}+9x_{9}\right)$$

$$\mu_{Y^{2}}=\frac{1}{N}\left(x_{1}+4x_{2}+9x_{3}+x_{4}+4x_{5}+9x_{6}+x_{7}+4x_{8}+9x_{9}\right)$$

Notably, the value of $g^{2}$ is equivalent to the standard metric $r^{2}$ under Hardy-Weinberg equilibrium. Similar alternative approaches have also been developed with good performance for long-range linkage disequilibrium and effective population size estimation from linkage disequilibrium patterns [21].

## 3 Results

**Fig. 2:**
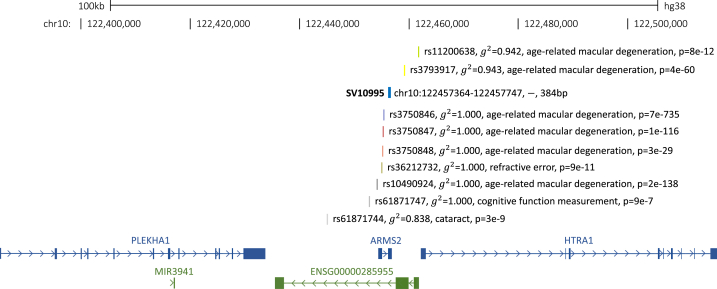
Example:*SV10995* and significant GWAS SNPs in high linkage disequilibrium.

Our analysis produced 16,377 GWAS-SNP-SV pairs in high linkage disequilibrium (g^2^ > 0.80) across 2355 traits from the GWAS catalog within the physical distance window. These SNP-SV pairs were composed of a total of 4715 unique SVs and 7892 unique GWAS-significant SNPs. The distribution of numbers of high linkage disequilibrium SNP-SV pairs for each chromosome is shown in Supplemental Table 1. To exemplify the utility of this resource, we show the findings for nine GWAS SNPs within the ±100kbp flanking region of SV10995 (insertion/deletion of 384 nucleotides) in the *PLEKHA1/ARMS2/HTRA1* region on chromosome 10q26 (Fig. 2). *PLEKHA1* encodes for the plecktrin homology domain containing A1 protein, which binds to phosphatidylinositol 3,4-bisphosphate. *ARMS2* encodes for the age-related maculopathy susceptibility 2 gene and is part of the choroidal extracellular matrix complex. *HTRA1* encodes for insulin growth factor binding serine peptidase 1. This region is a well-established age-related macular degeneration (AMD) susceptibility locus with a complex genetic framework [22,23]. Six of these 10q26-linked SNPs, rs11200638, rs3793917, rs3750846, rs3750847, rs3750848, and rs10490924 were all previously found to be highly associated with age-related macular degeneration (AMD) [[22], [23], [24], [25], [26], [27], [28], [29], [30], [31], [32], [33], [34], [35], [36], [37]]. The positions of these six SNPs in relation to the genes on chromosome 10q26 are displayed in Fig. 2. Four of these six SNPs were found to be in perfect linkage disequilibrium with SV10995 within the HGSVC sample set and rs11200638 and rs3793917 exhibited a $g^{2}=0.94$. Outside of AMD, rs61871747 was suggestively associated with cognitive function (p = 9.13E-07) [38], rs36212732 and rs10490924 were found to significantly correlate with refractive error [39,40], and rs61871744 was significantly associated with cataract [41]. SV10995 resides immediately (11bp (gene body)/431bp (cds)) downstream of *ARMS2*. It is possible that SV10995 affects the expression of ARMS2, which may, in turn, modify the risk for AMD. Interestingly, a different SV [42] was found to both be associated with AMD and have substantial effects on *ARMS2* mRNA stability [43].

**Fig. 3:**
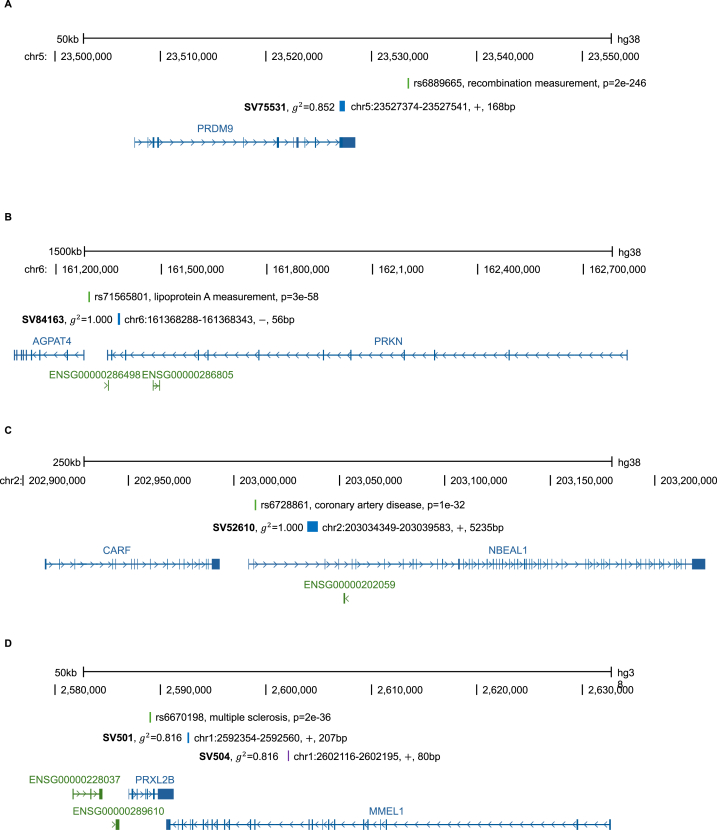
Four examples of structural variants in linkage disequilibrium with significant GWAS SNPs.

Further examples of SVs are illustrated in Fig. 3. We highlight instances where (1) an SV in LD potentially identifies a gene previously devoid of significant GWAS variants, (2) an SV may exhibit more substantial functional importance than the significant GWAS SNP originally reported, or (3) an SV in LD encompasses both aforementioned cases. We present examples across four different traits to illustrate the utility of this work: recombination hotspot activity, lipoprotein (a) levels, coronary artery disease, and multiple sclerosis.

### 3.1 SV755331 and recombination hotspot activity in the PRDM9 gene (Fig. 3a)

SV755331 is localized within LD with rs6889665 exhibiting a highly significant association with recombination hotspot activity (p-value = 2E-246) [44]. Notably, rs6889665 is located roughly 4 kilobases downstream of the PR domain-containing protein 9 (*PRDM9*) gene having a role in genetic recombination [45]. Given the predicted role of PRDM9 in orchestrating approximately 2500 recombination hotspots within individuals of West African ancestry (a stark contrast to its near inactivity in Europeans) [44], the presence of SV755331 within the PRDM9 coding region may exert consequential effects on the determination of recombination hotspots and/or recombination activities.

### 3.2 SV84163 in the context of lipoprotein (a) levels and the PRKN gene (Fig. 3b)

SV84163 is localized within LD with rs71565801 on chromosome 6. The association of rs71565801 with lipoprotein (a) levels has been demonstrated to be highly significant (p-value = 3E-58) [46]. The SNP is intergenically located between Parkin RBR E3 ubiquitin protein ligase (*PRKN)* and 1-acylglycerol-3-phosphate O-acyltransferase 4 (*AGPAT4*), 40 kb downstream of *PRKN* and 20 kb upstream of *AGPAT4*. While rs71565801 may be part of a gene regulatory sequence, SV84163 is specifically localized in the intronic region of *PRKN*, suggesting a potential regulatory function. It is noteworthy that liver-specific *Prkn* knockout mice demonstrate a predisposition to diet-induced hepatic steatosis, potentially due to dysregulated lipid metabolism [47]. Moreover, PRKN is implicated in the regulation of neuronal lipid homeostasis via the SREBP2-lipoprotein lipase pathway [48], which might influence lipid metabolism in other tissues. Collectively, these findings propose PRKN as a viable candidate for GWAS, justifying SV84163 as a genetic variant of interest.

### 3.3 SV52610 potential association with coronary artery disease and the NBEAL1 gene (Fig. 3c)

SV52610 is localized within LD with rs6728861 that is associated with coronary artery disease (p-value = 1E-32) on Chromosome 2 (Fig. 3c) [49]. While rs6728861 is located ∼6kbp upstream of the neurobeachin like 1 (*NBEAL1*) gene where strong functional relevance has not been shown, SV52610 is in the intronic region of *NBEAL1* and spans the putative *cis*-regulatory elements for gene expression of *NBEAL1* [[50], [51], [52]] (Fig. 3c). A recent study has shown that *NBEAL1* variants are correlated with NBEAL1 expression differences in artery tissue and an elevated risk of coronary artery disease in humans [53,54]. Additionally, NBEAL1 has been shown to regulate SREBP2 processing and cholesterol metabolism [53], suggesting that SV52610, located within the potential regulatory sequence of *NBEAL1*, could be considered a good candidate polymorphism affecting the gene expression of *NBEAL1* associated with coronary artery disease.

### 3.4 SV501 and SV504, multiple sclerosis, and MMEL1 (Fig. 3d)

Lastly, utilizing the linkage disequilibrium patterns between GWAS-significant SNPs and structural variants for fine mapping phenotype association signals may also reveal additional genes or regulatory motifs which are potentially causal. For example, a large two-staged study of multiple sclerosis susceptibility discovered a significant association with rs6670198 (p-value = 2E-36) [55]. Multiple sclerosis is a demyelination disease of the central nervous system driven by lymphocyte-mediated autoimmunity. Rs6670198 is an intronic SNP within PRXL2B on chr1p36, which encodes for the prostamide/prostaglandin F synthase protein. We found two new structural variants in high linkage disequilibrium with rs6670198: SV501 and SV504, both exhibiting $g^{2}$ values of 0.816 with rs6670198. Fig. 3d shows the position of both structural variants and the SNP with *PRXL2B*. SV501 is a 207bp insertion/deletion within intron 21 of the membrane metalloendopeptidase like 1-encoding gene, *MMEL1*. The enzyme is within the neural endopeptidase family. Interestingly, rs187786174 is located within SV501 and has been found to be associated with another autoimmune disease, rheumatoid arthritis, at a genome-wide significant level (p-value = 4E-14) [56]. SV504 is an 80bp insertion/deletion within intron 11 of *MMEL1*. Notably, intronic structural variants can disrupt gene regulation with substantial with higher average effect sizes than single nucleotide variants [57]. Both *PRXL2B* and *MMEL1* are reasonable candidates for causal functional effects on multiple sclerosis susceptibility. *MMEL1* is expressed in the central nervous system at the RNA level [58] and moderately expressed in lymphoid-derived cells at the protein level [59]. Protein-protein interaction analyses show that the membrane metallopeptidase like protein has strong interactions with several established neurodegeneration pathways, including signaling through the mineralocorticoid receptor (*NR3C2*) which has been implicated in neuroautoimmunity [60,61]. A study designed to evaluate multiple sclerosis GWAS signals in replication sample sets, researchers reported rs3748816 in *MMEL1* as conferring a predisposing effects [62]. In a large proteome study utilizing Mendelian randomization which included cerebral spinal fluid samples, increased levels of MMEL1 protein was found to have significant effects on inflating multiple sclerosis risk, suggesting a causal role of *MMEL1* in multiple sclerosis pathogenesis [63]. Hence, both *PRXL2B* and *MMEL1* are plausible multiple sclerosis risk genes, and with the discovery of SV501 and SV504 within *MMEL1* being highly correlated with the *PRXL2B*-linked rs6670198, further exploration of functional effects of these structural variants may be warranted.

## 4 Discussion

Through the creation of this repository of disease-associated SNPs that are highly correlated with newly discovered SVs, this archive can serve as an important resource for fine mapping causal variants in medically important traits. As SVs are substantial genomic changes which can modify gene expression and function and many have previously been shown to confer pathogenic effects, we anticipate that in some instances SVs in linkage disequilibrium with genome-wide significant SNPs may play a causal role in certain diseases.

In this study, we have used a threshold of g^2^ > 0.80 to define SNP-SV pairs in high linkage disequilibrium. Notably, a squared Pearson's correlation coefficient of 0.80 is considered the consensus threshold for tagging SNPs [64] and Halldorsson et al. showed that this threshold produced good results for capturing haplotype diversity across the genome [65]. While SVs exhibiting high linkage disequilibrium with reported genome-wide significant SNPs are reasonable candidates for causal effects, lower linkage disequilibrium values can also be important. As reported by Maadooliat et al. there is a simple relationship between (i) the disease association Chi-squared statistic at a biallelic causal site from a case/control study, (ii) the Chi-squared statistic testing for disease association at a site in linkage disequilibrium with the causal site, and (ii) the linkage disequilibrium value between the causal and marker sites [19]. In some instances where the effect size is large at the causal site (presumed to be the SV in this discussion), the allele frequency is sufficiently large, and there are sufficiently large numbers of samples, it may be reasonable to explore lower values of g^2^. In situations where there are SVs exhibiting high linkage disequilibrium with established genome-wide significant SNPs, then these should be considered excellent candidates for genotyping studies and, if significantly associated with disease, functional studies.

To advance research in the investigation of correlated SV-SNP pairs in the genome and identification of specific SVs that are candidates for causal effects, several future directions can be pursued: 1) As the goal is to identify likely causal SVs in regions that exhibit SNP disease association within GWAS, the development of new correlation measures specifically designed to harness signal from causal sites could accelerate the discovery of disease-causing SVs; 2) The SV data utilized in our database does not include all human SVs and is biased toward well-studied populations. As new SVs are discovered through long-read sequencing of large sample sets across different ancestries, these new SVs should be incorporated into databases with significant SNP data from ancestry-specific GWAS; and 3) The construction of an interactive database of these SV-SNP results would further enable the use of these findings from researchers in human disease genetics.

## Ethics approval and consent to participate

Not applicable.

## Consent for publication

Not applicable.

## Availability of data and materials

All data files including those detailing SV and GWAS SNP associations and results of GWAS-SNP-SV pairs are available at https://github.com/hliang-SchrodiLab/SV_SNPs.

## Funding

Research reported in this publication was supported by the 10.13039/100000053National Eye Institute of the 10.13039/100000002National Institutes of Health under the award number 10.13039/100000002NIH R01EY022086. Additional support provided by the 10.13039/100000092National Library of Medicine T15 LM007359, Computation and Informatics in Biology and Medicine training program. The content is solely the responsibility of the authors and does not necessarily represent the official views of the National Institutes of Health. Additional funding was received by the Timothy William Trout Chair in Eye Research and the 10.13039/100007015University of Wisconsin-Madison Center for Human Genomics and Precision Medicine.

Fig. 2 shows the region from chr10:122,357,364–122557747 (hg38 assembly) showing SV10995 (insertion/deletion structural variant), the ten SNPs associated with disease traits from the GWAS Catalog, the squared correlation ($g^{2})$ between each SNP and SV10995, and the p-value reported in the respective publication for each SNP. This information is displayed positionally in the context of *PLEKHA1*, the microRNA *MIR3941*, ENSG00000285955, *ARMS2*, and *HTRA1*.

Fig. 3 shows four examples of potentially functional structural variants exhibiting high linkage disequilibrium with SNPs that are highly significantly associated with traits. Fig. 3A shows the *PRDM9* region. Rs6889665 is highly associated with recombination rate and is located outside the *PRDM9* coding region. SV75531, a 168bp insertion/deletion structural variant in linkage disequilibrium with rs6889665, is a candidate for the disruption of PRDM9 function. In Fig. 3B, the chromosome 6 region strongly associated with Lp(a) levels at the intergenic SNP rs71565801, which is located between *AGPAT4* and *PRKN*. SV84163, within *PRKN*, is in complete linkage disequilibrium with rs71565801. The chromosome 2 region associated with coronary artery disease (rs6728861) is shown in Fig. 3C. SV52610, a 5235bp structural variant, is in complete linkage disequilibrium with rs6728861 and located within *NBEAL1*. Fig. 3D shows two structural variants, SV501 and SV504, within *MMEL1* which are both in high linkage disequilibrium with the multiple sclerosis SNP rs6670198 located within *PRXL2B*.

## CRediT authorship contribution statement

**Hao Liang:** Writing – review & editing, Writing – original draft, Software, Resources, Methodology, Investigation, Formal analysis, Data curation. **Joni C. Sedillo:** Writing – review & editing, Writing – original draft, Resources, Data curation. **Steven J. Schrodi:** Writing – review & editing, Writing – original draft, Supervision, Methodology, Investigation, Formal analysis, Conceptualization. **Akihiro Ikeda:** Writing – review & editing, Writing – original draft, Supervision, Funding acquisition, Conceptualization.

## Declaration of competing interest

The authors declare that they have no known competing financial interests or personal relationships that could have appeared to influence the work reported in this paper.
