# Supplementary material for: Structural variants in linkage disequilibrium with GWAS-significant SNPs
Source: Heliyon. 2024 May 28;10(11):e32053. doi: 10.1016/j.heliyon.2024.e32053 (PMC11177133; doi:10.1016/j.heliyon.2024.e32053)
Supplement: Multimedia component 1 [file mmc1.docx]

**Supplemental Table 1**

The number of genome-wide significant SNPs in high linkage disequilibrium ($g^{2}>0.80$ ) with a structural variant listed by chromosome.

| Chromosome | SNP-SV Pairs |
| --- | --- |
| chr1 | **1014** |
| chr2 | **1391** |
| chr3 | **946** |
| chr4 | **815** |
| chr5 | **801** |
| chr6 | **2087** |
| chr7 | **859** |
| chr8 | **771** |
| chr9 | **795** |
| chr10 | **552** |
| chr11 | **694** |
| chr12 | **689** |
| chr13 | **385** |
| chr14 | **257** |
| chr15 | **310** |
| chr16 | **747** |
| chr17 | **1095** |
| chr18 | **240** |
| chr19 | **751** |
| chr20 | **519** |
| chr21 | **153** |
| chr22 | **300** |
| chrX | **67** |
